# How (not) to mobilize health workers in the fight against vaccine hesitancy: Experimental evidence from Germany’s AstraZeneca controversy

**DOI:** 10.1186/s12889-022-12725-9

**Published:** 2022-03-16

**Authors:** Jan Priebe, Henning Silber, Christoph Beuthner, Steffen Pötzschke

**Affiliations:** 1 Bernhard Nocht Institute for Tropical Medicine, Bernhard-Nocht-Straße 74, Hamburg, 20359 Germany; 2 GESIS – Leibniz Institute for the Social Sciences, B6 4-5, Mannheim, 68159 Germany

**Keywords:** COVID-19, Misinformation, Vaccine hesitancy, Germany

## Abstract

**Background:**

COVID-19 vaccine hesistancy is a serious policy issue in Germany as vaccinations have stagnated at low levels compared to most other European countries. In this context, we study whether and how health workers can be leveraged to promote the COVID-19 vaccination campaign.

**Methods:**

We employed an information experiment with health workers in Germany to quantify how access to information related to (i) AstraZeneca’s vaccine safety, (ii) misinformation, (iii) individual health risks, and (iv) public health risks can sway health workers’ recommendations for any of the following vaccines: AstraZeneca, Johnson & Johnson, Moderna, Pfizer/BioNTech, Sinopharm, and Sputnik-V. The information experiment was conducted as a randomized controlled trial with four treatment arms and was embedded in an online survey.

**Results:**

Health workers reduce their willingness to recommend four out of six vaccines once they learn about different statements of European and German health authorities with respect to the safety of the AstraZeneca vaccine. Consistent with the discussion on AstraZeneca’s safety focusing on possible side effects among younger women, we find that especially female health workers become less likely to recommend the majority of COVID-19 vaccines. Lastly, we show that health workers vaccine recommendations are not affected by misinformation and appeals to individual or public health.

**Conclusion:**

In order to mobilize health workers in the fight against vaccine hesitancy, information campaigns need to be tailor-made for the target audience. In particular, health workers react to different types of information than the general public. As with the general public, we provide suggestive evidence that health workers require unambigious messages from drug authorities in order to support vaccination efforts. We believe that a more coordinated and coherent approach of public authorities can reduce the amount of mixed signals that health workers receive and therefore contribute to health workers engagement in the outroll of mass COVID-19 vaccination campaigns.

**Trial registration:**

The trial was registered retrospectively with the repository of the Open Science Framework (OSF) under the number osf.io/qa4n2.

## Introduction

The success of COVID-19 vaccination campaigns depends on the fast and widespread uptake of the vaccines among the general public. With different types of vaccines becoming increasingly available in developed countries, the policy focus is shifting toward demand-side constraints.

A particular concern relates to widespread COVID-19 vaccine hesitancy [1–4] as it is estimated that herd immunity can only be reached if between 55 percent to 85 percent of the population is vaccinated against COVID-19 [5, 6]. To address vaccine hesitancy and increase vaccination rates, governments frequently rely on information campaigns that deploy various individuals (health experts, celebrities, religious leaders), channels (media, health centres, religious institutions), and topics (the risks of the virus and the safety of the vaccines). Whether these campaigns are effective will depend on issues of access and trust. For instance, some people might not be reached through conventional media outreach campaigns, others might distrust the government and therefore disregard vaccinations [7–12].

In this context, health workers such as nurses, paramedics, and doctors are important actors. They have direct access to patients, relevant experience, and are often considered highly trustworthy [13]. In this way they are in a position to provide informal advice that can influence their patients, friends, family and the wider public to take-up COVID-19 vaccines [14].

Yet little is known about the type of vaccine recommendations that health workers give. Informal advice might differ from public recommendations, for a number of reasons. First, health workers are a highly selective population that (i) has an intrinsic interest in health topics, (ii) received more extensive training on the benefits and risks of vaccines, and (iii) is at high risk of catching COVID due to their work on the frontline [15]. Second, health workers - like anyone else - are exposed to multiple sources of information when forming their own opinions. As such, they can be affected by misinformation, selective information processing, and the public debate in general.

This paper aims to assess to what extent health workers are responsive to different types of health-related information. More specifically, our study asks several policy-relevant questions: Which COVID-19 vaccines do health workers prefer? How are vaccine recommendations of health workers influenced by information provision? Does information provision alter beliefs and intended behaviour beyond vaccine recommendations?

To provide associative empirical evidence on our research questions, we employ an information treatment experiment that alters the information set. The setting for our experiment is Germany. Between April and May 2021, we recruited via Facebook and Instagram 3,318 health workers for an online survey. As part of the survey health workers were randomized into one of five experimental groups and subsequently asked to state their willingness to recommend any of the following six vaccines: AstraZeneca, Johnson & Johnson, Moderna, Pfizer/BioNTech, Sinopharm, and Sputnik-V.

This paper proceeds in six sections. Country context section provides background information on the German context. Research design section describes our data. Main results section outlines the empirical strategy, presents the main results, and explores robustness checks. Discussion section discusses extensions to the main findings. Conclusion section offers concluding thoughts.

## Country context

The online survey was conducted during Germany’s “third wave” of COVID-19 infections that had about 22,000 new COVID-19 cases per day.^1^ At the time of the survey four vaccines were officially approved by European and German authorities for adults above 18 years: two vector (AstraZeneca, Johnson & Johnson) and two mRNA (Moderna, Pfizer/BioNTech) vaccines.^2^ By mid-April 2021 about 6 percent of the general population in Germany were fully vaccinated against COVID-19 and another 9.8 percent of the population had already received the first injection [16].^3^ In mid-April 2021 the majority of these people were vaccinated with Pfizer/BioNTech (about 67 percent) followed by AstraZeneca (about 22.5 percent), Moderna (about 9 percent), and Johnson & Johnson (about 0.9 percent).

COVID-19 related vaccine hesitancy is a relevant policy issue in Germany and is vaccine-specific. In general preferences for mRNA vaccines are stronger than those for vector vaccines. According to a survey conducted in March/April 2021 by Germany’s Robert Koch Institute about 23.2 percent (44.3 percent) of respondents declared that they were undecided or against being vaccinated with a mRNA (vector) vaccine [16].

The gap in acceptance rates between mRNA and vector vaccines is largely driven by views on the vaccines that are most commonly available in Germany – AstraZeneca and Pfizer/BioNTech. While acceptance rates are positively correlated with official vaccine efficacy rates (82 percent for AstraZeneca vs. 95 percent for Pfizer/BioNTech)^4^, a number of context-specific factors need to be considered. First, BioNTech is locally-based and the marketing of the vaccine in Germany emphasizes pride and trust into a ‘German vaccine’. Second, messages of national public health authorities were particular ambiguous with respect to AstraZeneca which increased distrust into the safety of the vaccine.^5^

## Research design

### Data, sample, and measures

We collected data during April and May 2021. Respondents were recruited via advertisements on Facebook and Instagram which linked to an online survey hosted on Unipark. Respondents did not receive any monetary incentives to participate in the survey.

As described in Table 1 the survey started with a short explanation concerning its purpose, highlighted the target group (health workers), and asked for informed consent (step 1). The survey took about 25 minutes to complete. 4,921 respondents finished steps 1 to 4 (see Table 1).

**Table 1:** Description of intervention arms

Step	Topic	Description
1	Introduction	Filter and survey background, objectives and consent
2	General information	Socio-demographics, personality measures
3	Information Treatment	Resp. received 1/5 possible information treatments
4	Outcomes & mechanisms	Willingness to recommend vaccines, measures on trust and perception of future

In step 2 of the survey, we collected information on the respondent’s socio-demographic and professional background. Likewise, we gathered data on the respondents own health, (i) whether the respondent had already received a COVID-19 vaccine, and (ii) whether the respondent already had an appointment for a vaccination (if not yet vaccinated).

The information treatment (step 3) was conducted with respondents who self-identified as health worker and who already received at least 1 COVID-19 vaccination. In total, 3,318 respondents (about 67 percent of the survey sample) matched these criteria.

In step 4 of the survey information on our principal outcome variables were collected. Respondents were asked to what extent they would recommend to a friend/family member of their own age and gender any of the following vaccines: AstraZeneca, Johnson & Johnson, Moderna, Pfizer/BioNTech, Sinopharm, and Sputnik V. Respondents had to state their preference for each of these vaccines using a 7-point Likert scale that ranged from 1 (not likely to recommend) to 7 (very likely to recommend). The order in which the recommendation for a particular vaccine was elicited was randomized.

Furthermore, step 4 gathered information on respondents’ trust into different government institutions and about respondents’ predictions regarding the COVID-19 situation in Germany in October 2021 (about six months after the survey).

### Intervention

Information treatments were conducted at step 3 of the online questionnaire. Once respondents reached step 3 a text was shown to them that consisted of about 2 paragraphs (about 4-5 sentences) with a specific theme. Respondents were randomized at the individual level into one of five groups for which the exact wording is depicted in Table 4 in the Appendix. The five groups can be summarized as follows:

**Treatment 1: Scientific AstraZeneca (AZ) debate** Subjects were exposed to the arguments and debate that led to well-established German drug regulators halting vaccinations with AstraZeneca, with the European Medicine Agency (EMA) later reiterating the advantages and benefits of the vaccine.

**Treatment 2: Misinformation** Subjects received information which highlighted arguments typically used by vaccine opponents.

**Treatment 3: Own health** Subjects received scientific information about the possible negative health consequences of a COVID-19 infection. It was highlighted that vaccinations could reduce the likelihood that the subject suffered severe diseases.

**Treatment 4: Public health** Subjects were informed about the rapid spread of COVID-19 in Germany. The aggressive transmission and the possible severe consequences for other people’s health - in particular the elderly - were highlighted.

**Control condition:** We implemented a ‘passive’ control group design (Haarland et al.: Designing information provision experiments, forthcoming). Subjects received no additional information before being asked about their vaccine recommendations.

To facilitate the effectiveness and reliability of the experiment several precautionary design decisions were taken. To increase the understanding of treatment messages, the text that appeared on the website was displayed in an easy-to-read format by adjusting spaces, highlighting particular words, and adjusted to be properly displayed on any possible device. Moreover, to minimize concerns about experimenter demand effects the wording and language used in each treatment was neutral and tried to not involve any suggestive expressions. Likewise, the treatments avoided complicated expressions that some respondents might not have understood. Lastly, the survey (step 1) informed respondents that the research project has no commercial interests, that data is stored anonymously, and that the research project strictly complies with European and German GDPR regulations.

### Descriptive statistics

Table 2 depicts descriptive statistics regarding our sample of health workers with Table 5 in the Appendix containing a detailed description of key variables.

**Table 2:** Summary statistics

Variable	Mean	Median	SD	Min.	Max.	Obs.
	(1)	(2)	(3)	(4)	(5)	(6)
Age	37.11	35.00	13.26	17.00	65.00	3,318
Female	0.75	1.00	0.43	0.00	1.00	3,318
HH size	2.08	2.00	1.27	1.00	9.00	3,318
Lives with children	0.16	0.00	0.37	0.00	1.00	3,318
Edu1: Degree not completed	0.00	0.00	0.07	0.00	1.00	3,318
Edu2: Hauptschule degree	0.02	0.00	0.13	0.00	1.00	3,318
Edu3: Realschule degree	0.27	0.00	0.44	0.00	1.00	3,318
Edu4: More than Realschule degree	0.42	0.00	0.49	0.00	1.00	3,318
Edu5: Degree not stated	0.29	0.00	0.45	0.00	1.00	3,318
Job: Doctor	0.04	0.00	0.20	0.00	1.00	3,318
Job: Nurse	0.61	1.00	0.49	0.00	1.00	3,318
Job: Paramedic	0.10	0.00	0.31	0.00	1.00	3,318
Job: Midwife	0.01	0.00	0.08	0.00	1.00	3,318
Job: Others	0.24	0.00	0.43	0.00	1.00	3,318
Willingness to take risk	4.21	4.00	1.28	0.00	7.00	3,318
Patience	6.67	7.00	2.53	1.00	11.00	3,318
Altruism	140.49	50.00	201.36	0.00	1000.00	3,276
COVID risk group	0.21	0.00	0.41	0.00	1.00	3,318
Received AZ	0.29	0.00	0.45	0.00	1.00	3,318
Received Moderna	0.00	0.00	0.00	0.00	0.00	3,318
Received J & J	0.07	0.00	0.26	0.00	1.00	3,318
Received Pfizer	0.64	1.00	0.48	0.00	1.00	3,318
Facebook	0.54	1.00	0.50	0.00	1.00	3,318
Smart phone	0.81	1.00	0.39	0.00	1.00	3,318

*Notes:* Sample consists of vaccinated health workers. Germany has a three tiered school system. After primary school (grade 1 to 4) children select into one of the following schools: Hauptschule (grade 5 to 9), Realschule (grade 5 to 10), and Gymnasium (grade 5 to 12/13). While the Hauptschule/Realschule track allows children to qualify for vocational trainings, the Gymnasium track in addition allows to apply for colleges and universities. Health workers were recruited via Facebook (54 percent of respondents) and Instagram (46 percent of respondents)

The analytical sample consists of 3,318 respondents. The average respondent is 37 years old and female (about 75 percent). The majority of respondents are nurses (about 61 percent) with a minority of respondents being medical doctors (about 4 percent), and paramedics (about 10 percent). Moreover, Table 2 shows the majority of respondents filled-out the survey via smartphone (about 81 percent) and were recruited via ads on Facebook (about 53 percent) and Instragram (about 47 percent). Furthermore, as shown Table 6 in the Appendix vaccinated health workers tend to be relatively younger, more female, and more risk averse compared to health workers that were not yet vaccined at the time of the survey.

Furthermore, most health workers had been vaccinated with Pfizer/BionTech (about 64 percent), followed by AstraZeneca (about 29 percent), and Johnson & Johnson (about 7 percent).

**Fig. 1:**
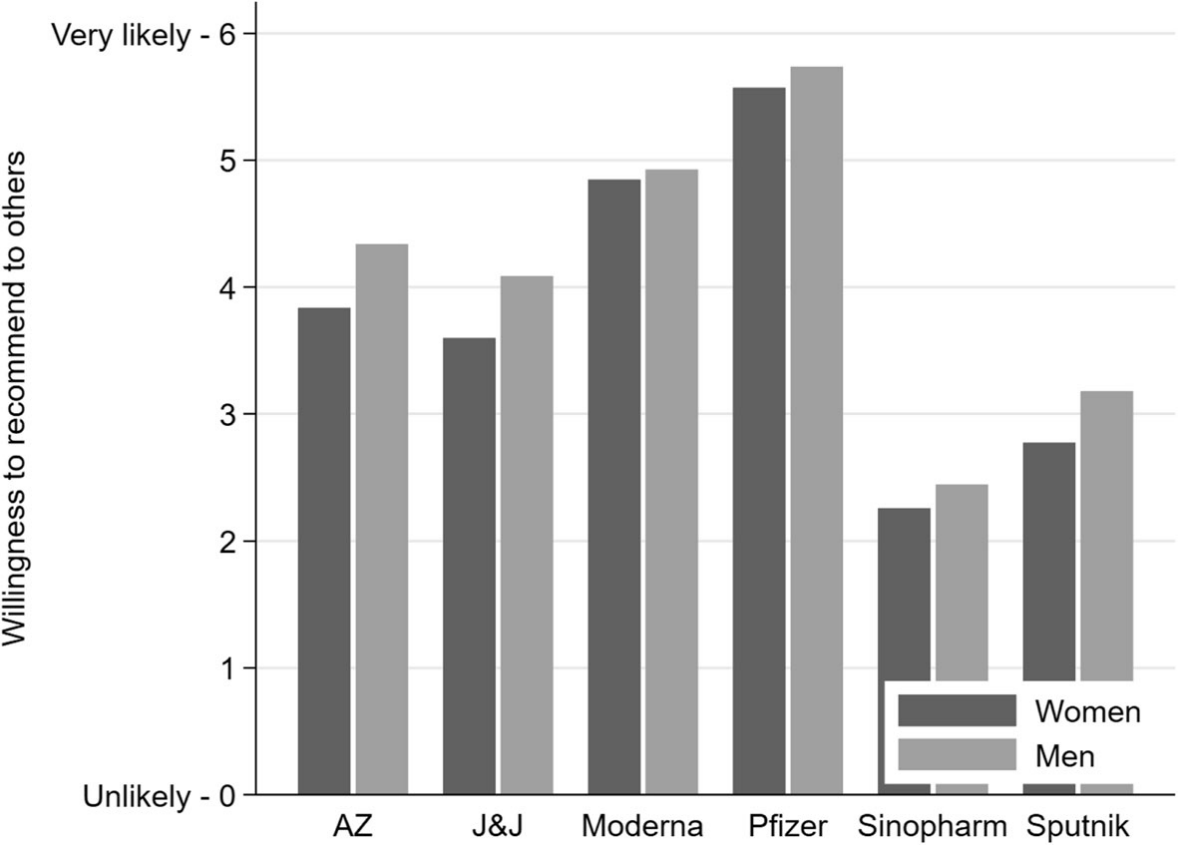
Willingness to recommend vaccines to others (control group only)

To assess the willingness of health workers to recommend any of the six vaccines in the absence of our information treatments, we provide in Fig. 1 descriptive statistics by gender for the control group. Among both men and women, the Pfizer/BioNTech vaccine was strongly recommended by almost all health workers, and the likelihood of recommending Moderna was also high. The willingness to recommend AstraZeneca and Johnson & Johnson was at a medium level. Finally, Sinopharm and Sputnik-V were least likely to be recommended. Men were slightly more likely to recommend five out of six vaccines. This effect was particularly visible for AstraZeneca, possibly due to the ongoing public debate in Germany that emphasises rare side effects of the vaccine among young women [19].

Furthermore, we provide results from balance tests in Table 7 in the Appendix. Out of the 126 comparisons (14 variables times 9 subgroup comparisons), only one is statistically significant at the five percent level (person has a completed college degree the comparison between T1 and the control group). Overall, we find that the randomization worked very well.^6^

## Main results

### Empirical specification

We estimate treatment effects by OLS based on the following regression model:

1 $$ Y_{is}=\alpha_{s}+ \sum_{r=1}^{r=4} \gamma_{r} T_{r} + X^{'}_{is}\beta + \epsilon_{is}  $$

where *Y*_*is*_ refers to the outcome variable (willingness to recommend vaccine) for individual *i* in province *s*, *α*_*s*_ indicate fixed effects for province s, and *X* refers to respondent-specific control variables.^7^*T*_*rj*_ are dummy variables indicating treatment arm *r*∈{1,2,3,4}. Robust standard errors are used.

### Results

Our main results are depicted in Table 3. Regarding the four information treatments we find the following: First, providing health workers with information on the reasoning and decisions of German and European drug regulating bodies with respect to AstraZeneca (T1) reduces health workers’ willingness to recommend AstraZeneca by 0.68 units. Second, obtaining information as part of T1 does not only affect recommendations with respect to AstraZeneca but creates negative spillovers to all other drugs that were less common/not yet approved at the time of the survey. While the willingness to recommend Johnson & Johnson, Sinopharm and Sputnik-V decreases, we observe an increase (albeit statistically insignificant) in recommending the vaccine of Pfizer/BioNTech.^8^ In addition, we observe a modest effect of exposing health workers to the arguments used by vaccine opponents and conspiracy theorists (T2). It appears that being exposed to these arguments rather reinforces health workers’ willingness to recommend vaccination. Moreover, our treatment arms that provide information on the relationship between COVID-19 and the respondent’s own or the public’s health (T3 & T4) have largely no effect on health workers’ willingness to recommend any of the six vaccines.

**Table 3:** Impact of treatments on willigness to recommend (OLS)

Parameter	AZ	J & J	Moderna	Pfizer	Sinopharm	Sputnik-V
	(1)	(2)	(3)	(4)	(5)	(6)
T1: Scientific AZ debate	-0.681	-0.314	-0.112	0.078	-0.492	-0.330
	(0.133)***	(0.128)**	(0.103)	(0.064)	(0.144)***	(0.140)**
T2: Misinformation	0.052	0.160	0.072	0.026	0.427	0.266
	(0.129)	(0.124)	(0.099)	(0.064)	(0.154)***	(0.140)*
T3: Own health	0.078	0.115	0.004	0.031	0.151	0.174
	(0.126)	(0.123)	(0.100)	(0.064)	(0.151)	(0.139)
T4: Public health	0.044	0.077	0.002	-0.007	0.152	0.263
	(0.128)	(0.124)	(0.100)	(0.065)	(0.151)	(0.139)*
Observations	3,318	3,318	3,318	3,318	3,318	3,318
r2	0.8141	0.8148	0.9112	0.9699	0.6120	0.7120
meanT0End	4.9624	4.7207	5.8669	6.6136	3.3068	3.8770
T1vsT2	0.0000	0.0002	0.0761	0.4228	0.0000	0.0000
T1vsT3	0.0000	0.0008	0.2697	0.4675	0.0000	0.0003
T1vsT4	0.0000	0.0023	0.2720	0.1960	0.0000	0.0000
T2vsT3	0.8376	0.7180	0.4956	0.9389	0.0741	0.5104
T2vsT4	0.9476	0.5058	0.4815	0.6164	0.0738	0.9840
T3vsT4	0.7843	0.7589	0.9863	0.5641	0.9952	0.5229
State FE	Yes	Yes	Yes	Yes	Yes	Yes
Basic Controls	Yes	Yes	Yes	Yes	Yes	Yes

*Notes:* ‘AZ’ refers to AstraZeneca, ‘J & J’ refers to Johnson & Johnson, and ‘Pfizer’ refers to Pfizer/BioNTech. State FE include controls for respondents place of residence (state). Basic controls include respondent’s age, gender, education, and survey specifics (order of vaccine-related questions, platform, device). Robust standard errors used. Standard errors are in parentheses. */**/*** denotes 10/5/1 percent significance levels

### Robustness checks

In order to assess, whether our previous findings are sensitive to our preferred empirical specification we show in the Appendix results from estimations that (i) include additional covariates (Table 8), (ii) alternative clustering of standard errors (Table 9), (iii) adjusting standard errors for multiple hypothesis testing (Table 10) following the procedure of [20, 21], and (iv) and a sample that focuses on nurses and therapists and therefore excludes medical doctors and administrative workers (Tables 11). Overall, our previous results remain.

## Discussion

The information treatment T1 seems to have contributed to reducing the willingness to recommend AstraZeneca and other less well-established COVID-19 vaccines. In the following, we consider possible mechanisms behind our finding and examine how in particular T1 affects health workers’ recommendations and beliefs.

### Gender

There are various reasons for why men and women might react differently to our information treatments. For instance, women tend to be slightly less likely to recommend COVID-19 vaccines and more likely to have joined demonstrations of vaccine opponents/conspiracy theories [22, 23], while possible side-effects of COVID-19 vaccines were more frequently discussed with respect to thrombosis among younger women [24].

**Fig. 2:**
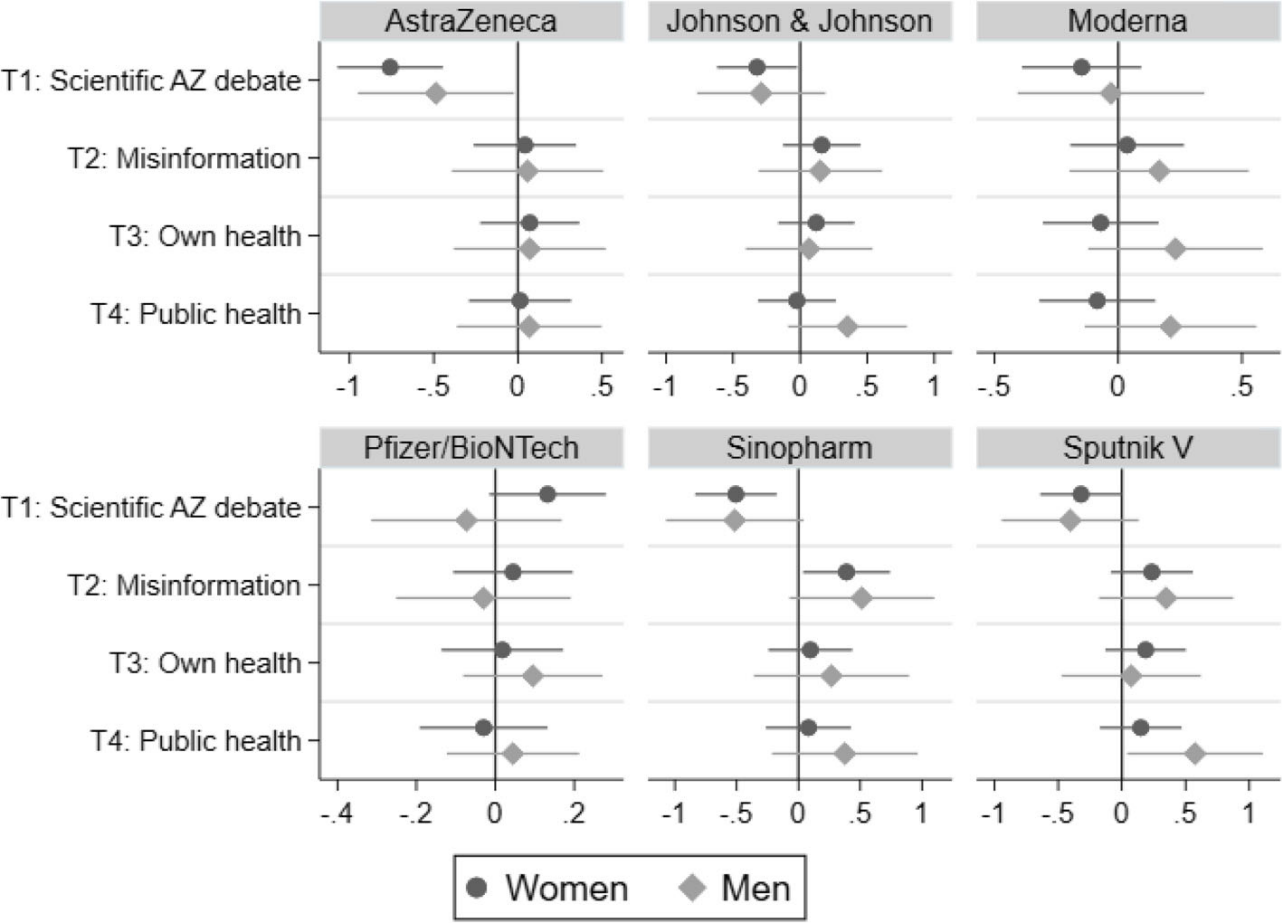
Impact of ITs on willingness to recommend vaccines to others (OLS). Note: Bars indicate 95% percentile intervals

Our results with respect to gender are summarized in Fig. 2 and Tables 12 and 13 in the online Appendix. For both, men and women, we find evidence for a negative impact of T1 on the willingness to recommend AstraZeneca. Likewise, for both genders, we observe negative spillovers of T1 to all less common/not yet approved vaccines. By and large, spillover effects appear to be more present among women; in particular with respect to the observed substitution effect away from AstraZeneca towards Pfizer/BioNTech.

### Economic preferences

**Fig. 3:**
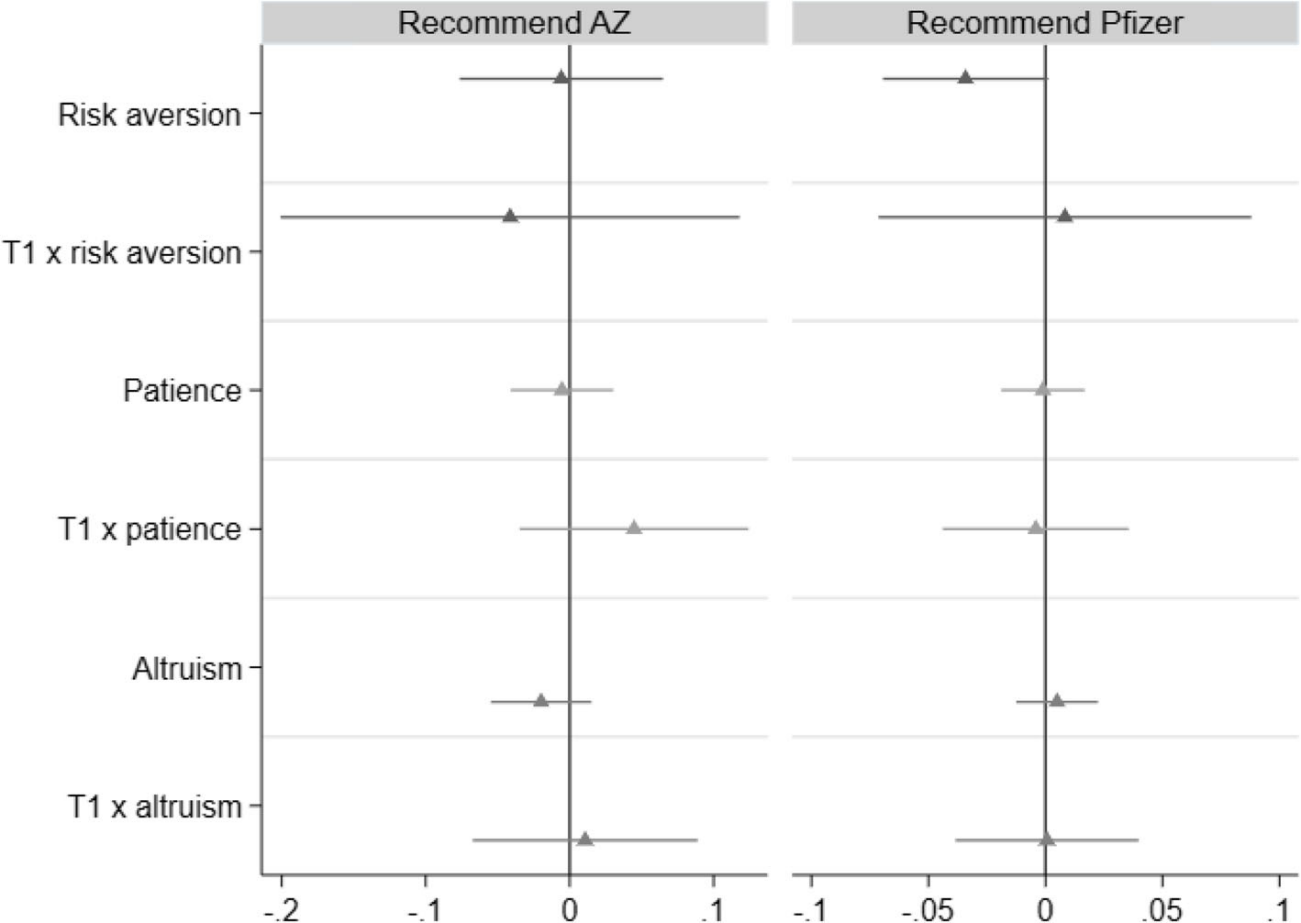
Impact of T1 and economic preferences on willingness to recommend vaccines to others (OLS). Note: Bars indicate 95% percentile intervals

It is well established that economic preferences such as risk, patience, and altruism can drive individual decision making. As part of our survey (step 2), we collected unincentivized preference measures based on survey items from the Global Preference Survey module [25]. In Fig. 3 below and Table 14 in the online Appendix, we investigate whether these preferences mediate the impact of T1. Therefore, we shed light on whether information related to AstraZeneca had a particularly strong impact among risk-averse health workers or those who are more patient, and less altruistic. By and large, we find that economic preferences neither influence the willingness to recommend a vaccine nor do these preferences mediate the impact of T1.

### Exposure

Health workers might react differently to information treatments and in particular to T1 depending on their individual exposure to COVID-19 risks and vaccines. In Table 15, we investigate whether respondents who belong to a high-risk group process T1 differently. Furthermore, we analyze whether the associative nature of the T1 relationship differs depending on whether a health worker was vaccinated with AstraZeneca vs. Pfizer/BioNTech.

**Fig. 4:**
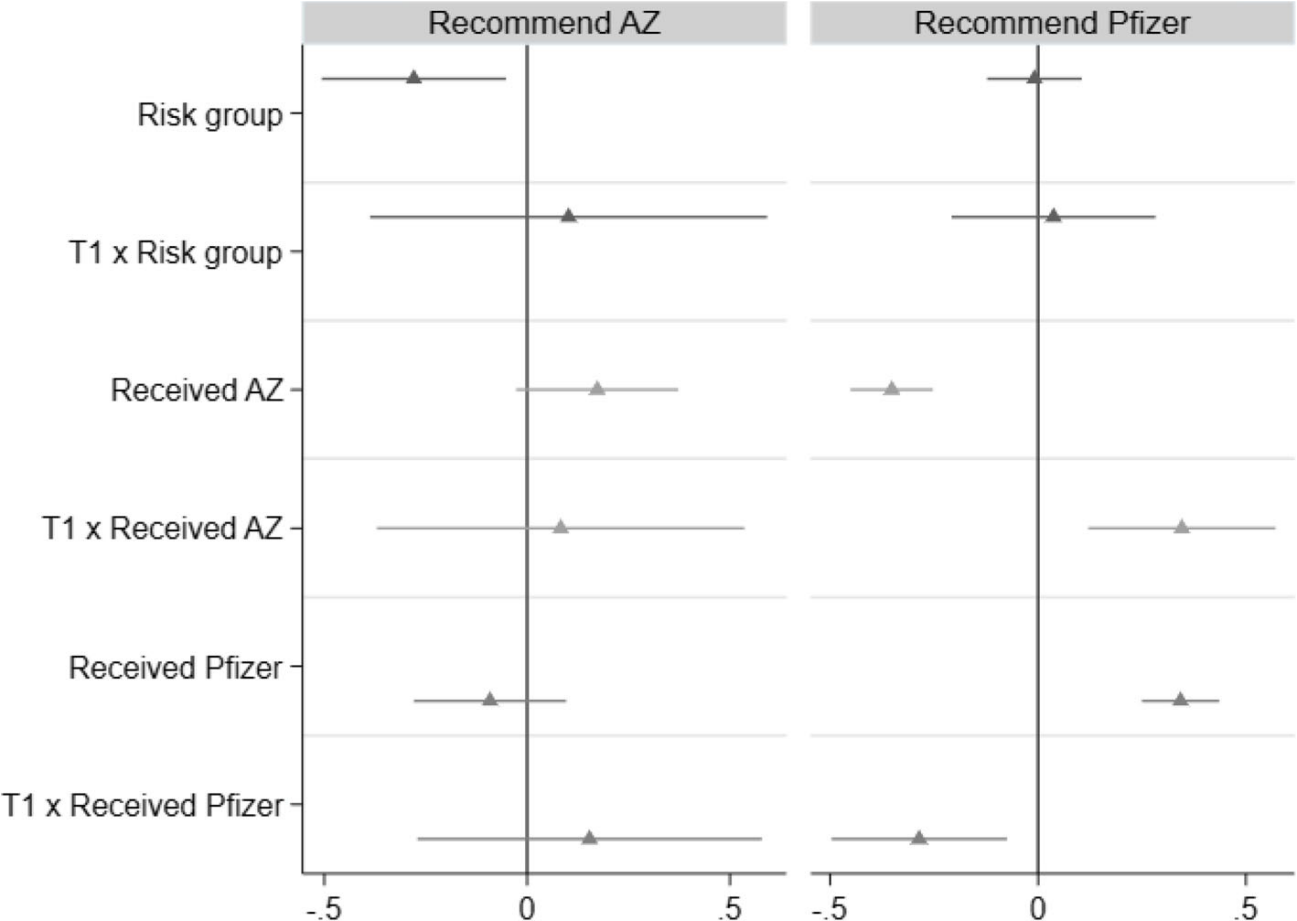
Impact of T1 and exposure on willingness to recommend vaccines to others (OLS). Note: Bars indicate 95% percentile intervals

As shown in Fig. 4 below and Table 15 in the online Appendix, we do not observe that a person’s COVID-19 risk status changes recommendations or the role of T1 (interaction effect). Regarding a person’s own COVID-19 vaccine history, we find that health workers who were vaccinated with AstraZeneca (Pfizer/BioNTech) were more likely to recommend the vaccine they were vaccinated with (panel A & B). Furthermore, we observe that particularly health workers who were vaccinated with AstraZeneca increase their willingness to recommend Pfizer/BioNTech once receiving the information from T1.

### Beliefs

Our experiment possibly alters the information set of health workers. Information can affect beliefs and (perceived) constraints which in turn can affect people’s choices. In this section we examine to what extent our information treatments have an impact on health workers’ satisfaction with government institutions and predictions about the future state of the world.

In Table 16 in the online Appendix we show the impact of our information treatments on health workers’ satisfaction with different government institutions at the state and national level (columns 1 to 4). In addition, we elicit satisfaction with Germany’s minister of health (at the time of the experiment), Mr. Jens Spahn, who featured very prominently in Germany’s COVID-19 strategy.

We find that three out of four information treatments (T2 & T3 & T4) seem to have no effect on satisfaction levels with the government. In contrast, we observe a sizeable positive impact of T1 on satisfaction with state-level institutions, such as the government and the ministry of health. While our data does not allow to disentangle mechanisms further, we believe that the results illustrate that the public and scientifically led discussion about the COVID-19 vaccine of AstraZeneca has re-ensured health workers that the government has a strict and reliable quality control mechanism in place. Moreover, the results suggest that the impact of T1 on vaccination recommendations is not driven by reductions in governmental trust; a channel highlighted in related contexts [26, 27].

In Table 17 in the online Appendix, we investigate whether our information treatments affected health worker’s attitudes and perception about the near future (reference period referred to October 2021; about six months after the survey). In columns 1 to 3, we focus on health workers’ predictions about how life in Germany will look like, while in columns 4-5 we look into future health-related behaviour in terms of the willingness to download COVID-19 apps (column 4) and refresh their own DPT vaccination when it is due.

Overall, we do not find that our information treatments affected health workers’ views of the future and their own predicted health behaviour.

## Conclusion

In this paper, we examine whether health workers’ views on COVID-19 vaccines can be swayed by information provision. Employing an information treatment experiment with four treatment arms and one control condition, we investigate whether health workers’ vaccine recommendations change once they learn from information related to (i) conspiracy theories, (ii) scientific discussions of drug authorities on AstraZeneca, (iii) risk of COVID-19 for their own health, and (iv) risk of COVID-19 for the public’s health. The experiment was conducted as part of an online survey with 3,318 health workers in April/May 2021 in Germany.

Our findings suggest that health workers’ willingness to recommend COVID-19 vaccines strongly depends on the messages conveyed by public drug regulators. More specifically, we find that the mixed messages that were conveyed by German and European authorities with respect to the vaccine of AstraZeneca decreased health workers’ acceptance of the vaccine. Moreover, we observe negative spillover effects to other in Germany less established vaccines such as Johnson & Johnson, Sinopharm, and Sputnik-V. Therefore, health workers became less likely to recommend those latter vaccines once learning about statements from the public drug authorities related to AstraZeneca. In contrast, we do not find that health workers’ vaccine recommendations are affected by misin- formation and arguments related to their own/the public’s health.

We believe that our experiment contributes to the existing literature in important ways. First, we relate to the literature that examines the impact of information provision on vaccination intentions and outcomes. While the literature traditionally had focused on routine vaccinations such as those for measles and influenza [28–30], a number of recent studies focused on the effect of information on attitudes towards COVID-19 vaccines [7, 31–33]. Second, we relate to the literature that studies the impact of information on a broader set of COVID-19 related outcomes such as stigmatization [34, 35] and beliefs about its risk factors and contagiousness [26, 36–38]. In contrast to both strands of the literature, our study does not focus on vaccine intentions among the general population but on vaccine recommendations of health workers. Consequently, our study closes an important gap in the existing literature by shedding light on how to best mobilize health workers in the global fight against vaccine hesitancy.

In this context we believe that our study entails important policy implications for the design of health campaigns. First, our results underscore that information campaigns need to be tailor-made with the specific target audience in mind. While information campaigns related to misinformation and/or appeals to individual/societal benefits had been shown to affect COVID-19 vaccination attitudes among the general population, we illustrate that such information does not affect health workers. Health workers, however, are nonetheless highly responsive to the information environment; in our setting information from public health authorities.

Second, we think that the negative impact of our AstraZeneca information treatment on the willingness to recommend several approved COVID-19 vaccines shows that public health authorities should coordinate public health messages more closely. The example that we selected for our study refers to the case in which within a very short time period (three days) the recommendation for the general public was revised two times (from being advisable, to being suspended, to being advisable again). While the public’s trust into the AstraZeneca vaccine never recovered from the related temporary suspension, we illustrate related impacts among health professionals.

Third, we highlight that policy makers need to carefully consider the consequences of emergency drug approval processes. While an early approval can save many lifes, it comes with the risk of revising recommendations and guidelines several times, which ultimately can substantially slow down vaccination campaigns over the medium to long-run.

## Appendix

**Table 4:** Description of intervention arms

Intervention	Description
Treatment 1	In Germany, the Paul Ehrlich Institute monitors the safety of vaccines and biomedical drugs. On March 15, 2021, the Paul Ehrlich Institute recommended the temporary suspension of vaccinations with AstraZeneca’s COVID-19 vaccine. There has been an increased incidence of rare cerebral venous thrombosis with the vaccine. On March 18, 2021, the European Medicines Agency (EMA) declared that AstraZeneca’s vaccine was safe and effective. However, the EMA recommended that there be greater awareness of risks and that these be included in the package. The decision of the Paul Ehrlich Institute and the EMA illustrate that there are pro and con arguments for vaccination.
Treatment 2	In recent weeks and months, numerous demonstrations have taken place regarding the Corona situation in Germany. These demonstrations address a variety of COVID-19 vaccination issues. Below we list some of the main statements made: 1. Several people have died from the COVID vaccine., 2. The vaccine is ineffective because people can still become infected after being vaccinated, 3. The vaccine damages genetic material, 4. The vaccine can cause cancer, 5. The vaccine can cause infertility 6. Speed took precedence over safety in the approval of the vaccine, 7. Many participants have died in the vaccine trials
Treatment 3	COVID-19 can cause asymptomatic, asymptomatic, or severe infections with pneumonia and other organ involvement, ranging from lung and multiple organ failure to death. A proportion of COVID-19 patients continue to struggle with effects weeks or months after the onset of infection. According to the current knowledge of the Robert Koch Institute, persons vaccinated against COVID-19 are more likely not to contract COVID-19 after contact with SARS-CoV-2. Accordingly, vaccinated persons have a higher probability of remaining healthy.
Treatment 4	According to the Robert Koch Institute (as of March 25, 2021), more than 2.7 million people in Germany have contracted COVID-19 to date and more than 65,000 people have died from COVID-19. Elderly people and people with chronic respiratory diseases have a higher probability of dying from COVID-19. Although some elderly persons have received SARS-CoV-2 vaccinations, the vaccinations ultimately do not provide absolute (100%) protection, so elderly/chronically ill persons remain at risk as long as high incidence rates continue to prevail in the general population. However, according to recent studies, between 75 and 95 percent of the population must be vaccinated or have natural antibodies to COVID-19 (e.g., from previous disease) for general protection (herd immunity) against COVID-19 to be assumed.

**Table 5:** Description of main variables

Variable	Type	Description
Outcome variables		
Vaccine recommendation	ordinal	Six ordinal variables (1 variable for each of the six vaccines) that capture the willingness to recommend vaccine to another person
		Response category: 7-point Likert scale from 1 (unlikely) to 7 (very likely)
Satisfaction (government)	ordinal	Satisfaction with five government institutions/person
		Response: 7-item Likert scale (1=very dissatisfied, 7=very satisfied
COVID-App	ordinal	Willingness to download COVID warning application to smartphone (0=unwilling, 1=maybe, 2=willing)
DPT-vaccine	ordinal	Willingness to refresh DPT (diphtheria, pertussis, and tetanus) vaccine in the future; (0=unwilling, 1=maybe, 2=willing)
Life in Germany	ordinal	Questions related to how life (cafes, travels) will look like in October 2021, Response: 7-point Likert scale, (1=very unlikely, 7=very likely)
Respondent characteristics		
Age	categorical	Age in years
Gender	binary	Gender of respondent (0=male, 1=female)
Education	binary	Dummy variables for the highest completed level of education (0=no, 1=yes); 4 variables for 5 different levels
Risk	ordinal	General risk question, Response is based on 7-point Likert scale (1=unwilling to take any risk,7=very willing to take risks)
Patience	ordinal	General patience question, Response is based on 10-point Likert scale, (1=very impatient, 10=very patient)
Altruism	categorical	Hypothetical lottery win. Respondents are asked to imagine to win 1,000 Euro in a lottery. How much of the win would they share with an unknown random person
		Response can be between 0 and 1,000 Euro
Risk group	binary	Belongs to COVID-19 health risk group (0=no, 1=yes)
Received vaccination	binary	Dummy variables indicating whether respondent was vaccinated with Pfizer/BioNTech or AstraZeneca (0=no, 1=yes)
Survey variables		
Platform	binary	Survey link from social media platform (0=instagram, 1=facebook)
Smart phone	binary	Survey was taken on smart phone (0=no, 1=yes)
Randomization order	binary	Five dummy variables indicating order of questions related to the recommendations for a specific vaccine (0=no, 1=yes)

*Notes:* Questions on risk, patience, and altruism were taken from the Global Preference Survey Module [25]

**Table 6:** Balance table: Respondent characteristics

	Mean values		Difference
Variable	No vaccine	Vaccine	(*p*-value)
Age	36.97	34.79	2.18***
	(13.27)	(12.54)	(0.00)
Female	0.75	0.81	-0.06***
	(0.43)	(0.39)	(0.00)
HH size	2.55	2.64	-0.09*
	(1.27)	(1.33)	(0.08)
Lives with children	0.18	0.26	-0.08***
	(0.38)	(0.44)	(0.00)
ob: Doctor	0.04	0.01	0.03***
	(0.20)	(0.12)	(0.00)
Job: Nurse	0.61	0.66	-0.05***
	(0.49)	(0.48)	(0.00)
Job: Paramedic	0.10	0.04	0.06***
	(0.30)	(0.19)	(0.00)
Job: Midwife	0.01	0.01	-0.00
	(0.08)	(0.09)	(0.58)
Job: Others	0.24	0.26	-0.03**
	(0.43)	(0.44)	(0.05)
Risk	4.20	4.34	-0.13***
	(1.28)	(1.34)	(0.00)
Patience	6.67	6.67	0.00
	(2.53)	(2.62)	(0.97)
Altruism	142.42	134.60	7.82
	(205.63)	(207.58)	(0.24)
Risk group	0.21	0.21	0.00
	(0.41)	(0.41)	(0.72)
Facebook	0.54	0.54	-0.00
	(0.50)	(0.50)	(0.94)
Smart phone	0.82	0.82	-0.00
	(0.39)	(0.39)	(0.86)
Observations	3,537	1,384	4,921

*Notes:* ‘No vaccine’ refers to health workers who had not yet received a COVID-19 vaccination. ‘Vaccine’ refers to health workers who already received at least 1 COVID-19 vaccine. */**/*** denotes significant at the 10/5/1 percent significance levels

**Table 7:** Balance Table: Respondent characteristics by treatment arm

	Mean values					Differences (*p*-value)									
Variable	C	T1	T2	T3	T4	C vs T1	C vs T2	C vs T3	C vs T4	T1 vs T2	T1 vs T3	T1 vs T4	T2 vs T3	T2 vs T4	T3 vs T4
	(1)	(2)	(3)	(4)	(5)	(6)	(7)	(8)	(9)	(10)	(11)	(12)	(13)	(14)	(15)
Age	37.88	36.51	37.17	37.23	37.23	-1.38*	-0.72	-0.65	-1.21*	-0.66	-0.73	-0.17	-0.07	0.49	0.56
	(13.58)	(13.02)	(13.37)	(13.38)	(13.38)	(0.06)	(0.33)	(0.37)	(0.10)	(0.37)	(0.32)	(0.82)	(0.93)	(0.50)	(0.44)
Female	0.75	0.73	0.74	0.78	0.78	-0.01	-0.00	0.03	-0.01	-0.01	-0.04*	-0.01	-0.03	0.00	0.04
	(0.44)	(0.44)	(0.44)	(0.42)	(0.42)	(0.57)	(0.90)	(0.19)	(0.80)	(0.67)	(0.06)	(0.76)	(0.15)	(0.90)	(0.12)
Edu1	0.00	0.00	0.01	0.00	0.00	0.00	0.01*	0.00	0.00	-0.00	0.00	-0.00	0.00	0.00	-0.00
	(0.04)	(0.07)	(0.09)	(0.05)	(0.05)	(0.28)	(0.09)	(0.56)	(0.16)	(0.49)	(0.60)	(0.73)	(0.23)	(0.72)	(0.39)
Edu2	0.02	0.02	0.02	0.01	0.01	-0.00	-0.00	-0.01	-0.00	0.00	0.00	-0.00	0.00	-0.00	-0.01
	(0.15)	(0.14)	(0.13)	(0.12)	(0.12)	(0.70)	(0.53)	(0.33)	(0.81)	(0.81)	(0.56)	(0.89)	(0.74)	(0.70)	(0.47)
Edu3	0.26	0.29	0.26	0.25	0.25	0.03	-0.00	-0.01	0.03	0.03	0.04	-0.00	0.01	-0.03	-0.04*
	(0.44)	(0.45)	(0.44)	(0.43)	(0.43)	(0.28)	(0.96)	(0.59)	(0.21)	(0.26)	(0.11)	(0.87)	(0.63)	(0.20)	(0.07)
Edu4	0.40	0.43	0.43	0.43	0.43	0.03	0.03	0.03	-0.00	-0.00	-0.01	0.03	-0.00	0.03	0.03
	(0.49)	(0.49)	(0.50)	(0.50)	(0.50)	(0.35)	(0.32)	(0.26)	(0.96)	(0.95)	(0.85)	(0.33)	(0.89)	(0.30)	(0.24)
Edu5	0.31	0.26	0.29	0.30	0.30	-0.05**	-0.03	-0.01	-0.03	-0.02	-0.04	-0.02	-0.02	0.01	0.02
	(0.46)	(0.44)	(0.45)	(0.46)	(0.46)	(0.04)	(0.28)	(0.63)	(0.20)	(0.32)	(0.11)	(0.42)	(0.55)	(0.84)	(0.42)
Risk	4.22	4.21	4.27	4.22	4.22	-0.01	0.05	-0.00	-0.08	-0.05	-0.00	0.07	0.05	0.13*	0.08
	(1.26)	(1.28)	(1.32)	(1.25)	(1.25)	(0.93)	(0.49)	(0.98)	(0.25)	(0.45)	(0.94)	(0.30)	(0.48)	(0.08)	(0.26)
Patience	6.73	6.56	6.63	6.66	6.66	-0.17	-0.10	-0.07	0.03	-0.07	-0.10	-0.20	-0.03	-0.13	-0.10
	(2.53)	(2.58)	(2.53)	(2.53)	(2.53)	(0.24)	(0.47)	(0.63)	(0.82)	(0.64)	(0.47)	(0.16)	(0.80)	(0.35)	(0.48)
Altruism	136.83	150.20	144.04	135.92	135.92	13.37	7.21	-0.91	-0.74	6.16	14.28	14.11	8.12	7.94	-0.17
	(196.72)	(209.47)	(204.69)	(208.10)	(208.10)	(0.23)	(0.51)	(0.93)	(0.94)	(0.60)	(0.22)	(0.20)	(0.48)	(0.47)	(0.99)
Risk group	0.21	0.22	0.19	0.23	0.23	0.01	-0.02	0.01	-0.01	0.03	-0.01	0.02	-0.04*	-0.02	0.02
	(0.41)	(0.42)	(0.39)	(0.42)	(0.42)	(0.69)	(0.27)	(0.52)	(0.76)	(0.14)	(0.81)	(0.49)	(0.08)	(0.43)	(0.34)
Facebook	0.56	0.53	0.54	0.54	0.54	-0.03	-0.02	-0.02	-0.02	-0.01	-0.01	-0.01	-0.00	0.00	0.00
	(0.50)	(0.50)	(0.50)	(0.50)	(0.50)	(0.27)	(0.46)	(0.55)	(0.45)	(0.73)	(0.61)	(0.74)	(0.88)	(0.99)	(0.87)
Smart phone	0.81	0.82	0.81	0.82	0.82	0.00	-0.01	0.00	-0.01	0.01	-0.00	0.01	-0.01	-0.00	0.01
	(0.39)	(0.39)	(0.39)	(0.39)	(0.39)	(0.96)	(0.77)	(0.87)	(0.80)	(0.74)	(0.92)	(0.77)	(0.66)	(0.97)	(0.68)
Observations	691	641	648	682	682	1,332	1,339	1,373	1,347	1,289	1,323	1,297	1,330	1,304	1,338

*Notes:* ‘C’ refers to control group, ‘T1’ to treatment arm 1, ‘T2’ to treatment arm 2, ‘T3’ to treatment arm 3, and ‘T4’ to treatment arm 4. Standard errors are in parentheses. */**/*** denotes significant at the 10/5/1 percent significance levels

**Table 8:** Impact of treatments on willingness to recommend COVID-19 vaccines (OLS): Extended controls

Parameter	AZ	J & J	Moderna	Pfizer	Sinopharm	Sputnik-V
	(1)	(2)	(3)	(4)	(5)	(6)
T1: Scientific AZ debate	-0.683	-0.329	-0.111	0.080	-0.504	-0.332
	(0.134)***	(0.130)**	(0.104)	(0.065)	(0.146)***	(0.141)**
T2: Misinformation	0.037	0.162	0.076	0.023	0.423	0.277
	(0.130)	(0.124)	(0.099)	(0.065)	(0.155)***	(0.140)**
T3: Own health	0.087	0.112	0.016	0.037	0.151	0.178
	(0.127)	(0.124)	(0.100)	(0.064)	(0.153)	(0.140)
T4: Public health	0.075	0.095	0.022	-0.002	0.162	0.288
	(0.128)	(0.124)	(0.098)	(0.065)	(0.152)	(0.140)**
Observations	3,276	3,276	3,276	3,276	3,276	3,276
r2	0.8167	0.8169	0.9134	0.9702	0.6131	0.7141
meanT0End	4.9624	4.7207	5.8669	6.6136	3.3068	3.8770
T1vsT2	0.0000	0.0002	0.0745	0.3902	0.0000	0.0000
T1vsT3	0.0000	0.0007	0.2301	0.5087	0.0000	0.0003
T1vsT4	0.0000	0.0010	0.1990	0.2158	0.0000	0.0000
T2vsT3	0.6956	0.6845	0.5489	0.8295	0.0807	0.4805
T2vsT4	0.7723	0.5892	0.5879	0.7082	0.0922	0.9358
T3vsT4	0.9208	0.8929	0.9476	0.5508	0.9462	0.4310
State FE	Yes	Yes	Yes	Yes	Yes	Yes
Basic Controls	Yes	Yes	Yes	Yes	Yes	Yes
Extended Controls	Yes	Yes	Yes	Yes	Yes	Yes

*Notes:* ‘AZ’ refers to AstraZeneca, ‘J & J’ refers to Johnson & Johnson, and ‘Pfizer’ refers to Pfizer/BioNTech. State FE include controls for respondents place of residence (state). Basic controls include respondent’s age, gender, education, and survey specifics (order of vaccine-related questions, platform, device). Extended controls refers to additional controls on respondent’s preferences (risk, patience, altruism) and whether she/he had ever been diagnosed with Covid. Robust standard errors used. Standard errors are in parentheses. */**/*** denotes 10/5/1 percent significance levels

**Table 9:** Impact of treatments on willingness to recommend COVID-19 vaccines (OLS): Alternative clustering

Parameter	AZ	J & J	Moderna	Pfizer	Sinopharm	Sputnik-V
	(1)	(2)	(3)	(4)	(5)	(6)
T1: Scientific AZ debate	-0.681	-0.314	-0.112	0.078	-0.492	-0.330
	(0.129)***	(0.125)**	(0.099)	(0.060)	(0.140)***	(0.146)**
T2: Misinformation	0.052	0.160	0.072	0.026	0.427	0.266
	(0.115)	(0.090)*	(0.085)	(0.092)	(0.155)**	(0.133)*
T3: Own health	0.078	0.115	0.004	0.031	0.151	0.174
	(0.127)	(0.129)	(0.098)	(0.054)	(0.090)	(0.155)
T4: Public health	0.044	0.077	0.002	-0.007	0.152	0.263
	(0.116)	(0.126)	(0.059)	(0.078)	(0.141)	(0.103)**
Observations	3,318	3,318	3,318	3,318	3,318	3,318
r2	0.8141	0.8148	0.9112	0.9699	0.6120	0.7120
meanT0End	4.9624	4.7207	5.8669	6.6136	3.3068	3.8770
T1vsT2	0.0000	0.0045	0.0420	0.3879	0.0000	0.0041
T1vsT3	0.0000	0.0003	0.1064	0.4283	0.0000	0.0012
T1vsT4	0.0000	0.0014	0.2067	0.0918	0.0000	0.0000
T2vsT3	0.8037	0.7162	0.3708	0.9455	0.0352	0.5353
T2vsT4	0.9541	0.4893	0.4958	0.5962	0.0883	0.9853
T3vsT4	0.7233	0.6302	0.9852	0.5123	0.9923	0.5182
State FE	Yes	Yes	Yes	Yes	Yes	Yes
Basic Controls	Yes	Yes	Yes	Yes	Yes	Yes

*Notes:* ‘AZ’ refers to AstraZeneca, ‘J & J’ refers to Johnson & Johnson, and ‘Pfizer’ refers to Pfizer/BioNTech. State FE include controls for respondents place of residence (state). Basic controls include respondent’s age, gender, education, and survey specifics (order of vaccine-related questions, platform, device). Standard errors are clustered at the state level. Standard errors are in parentheses. */**/*** denotes 10/5/1 percent significance levels

**Table 10:** Adjusted *p*-values for multiple hypothesis testing

Parameter	AZ	J & J	Moderna	Pfizer	Sinopharm	Sputnik-V
	(1)	(2)	(3)	(4)	(5)	(6)
Full Sample						
T1	0.000	0.045	0.392	0.392	0.005	0.054
T2	0.901	0.519	0.835	0.901	0.023	0.231
T3	0.890	0.810	0.966	0.890	0.810	0.685
T4	0.977	0.929	0.992	0.992	0.806	0.268
Women Sample						
T1	0.000	0.108	0.228	0.165	0.020	0.139
T2	0.937	0.663	0.937	0.896	0.121	0.491
T3	0.947	0.895	0.947	0.947	0.947	0.728
T4	0.981	0.981	0.942	0.977	0.976	0.890
Men Sample						
T1	0.210	0.552	0.889	0.768	0.352	0.464
T2	0.946	0.868	0.790	0.946	0.357	0.614
T3	0.987	0.987	0.679	0.854	0.854	0.987
T4	0.865	0.407	0.516	0.865	0.516	0.139
State FE	Yes	Yes	Yes	Yes	Yes	Yes
Basic Controls	Yes	Yes	Yes	Yes	Yes	Yes

*Notes:* ‘AZ’ refers to AstraZeneca, ‘J & J’ refers to Johnson & Johnson, and ‘Pfizer’ refers to Pfizer/BioNTech. State FE include controls for respondents place of residence (state). Basic controls include respondent’s age, gender, education, and survey specifics (order of vaccine-related questions, platform, device). *P*-value adjustments were calculated based on 2,000 replications using the RWOLF package in STATA

**Table 11:** Impact of treatments on willingness to recommend COVID-19 vaccines (OLS): Sample without doctors

Parameter	AZ	J & J	Moderna	Pfizer	Sinopharm	Sputnik-V
	(1)	(2)	(3)	(4)	(5)	(6)
T1: Scientific AZ debate	-0.783	-0.362	-0.117	0.046	-0.488	-0.328
	(0.143)***	(0.138)***	(0.111)	(0.070)	(0.155)***	(0.150)**
T2: Misinformation	0.012	0.091	0.035	0.015	0.424	0.256
	(0.138)	(0.134)	(0.107)	(0.069)	(0.164)***	(0.149)*
T3: Own health	-0.009	0.001	-0.006	0.008	0.047	0.088
	(0.136)	(0.132)	(0.107)	(0.069)	(0.161)	(0.148)
T4: Public health	0.003	0.027	-0.033	-0.020	0.153	0.249
	(0.138)	(0.132)	(0.107)	(0.070)	(0.161)	(0.149)*
Observations	2,906	2,906	2,906	2,906	2,906	2,906
r2	0.8113	0.8113	0.9093	0.9682	0.6102	0.7098
meanT0End	4.9624	4.7207	5.8669	6.6136	3.3068	3.8770
T1vsT2	0.0000	0.0012	0.1757	0.6598	0.0000	0.0001
T1vsT3	0.0000	0.0091	0.3254	0.5957	0.0006	0.0056
T1vsT4	0.0000	0.0047	0.4518	0.3605	0.0000	0.0001
T2vsT3	0.8795	0.5015	0.7070	0.9240	0.0215	0.2632
T2vsT4	0.9495	0.6349	0.5296	0.6218	0.0987	0.9631
T3vsT4	0.9297	0.8399	0.8049	0.6961	0.5099	0.2818
State FE	Yes	Yes	Yes	Yes	Yes	Yes
Basic Controls	Yes	Yes	Yes	Yes	Yes	Yes

**Table 12:** Women: Impact of treatments on willingness to recommend COVID-19 vaccines (OLS)

Parameter	AZ	J & J	Moderna	Pfizer	Sinopharm	Sputnik-V
	(1)	(2)	(3)	(4)	(5)	(6)
T1: Scientific AZ debate	-0.759	-0.321	-0.148	0.132	-0.506	-0.321
	(0.160)***	(0.152)**	(0.123)	(0.075)*	(0.168)***	(0.163)**
T2: Misinformation	0.042	0.161	0.036	0.045	0.389	0.235
	(0.155)	(0.147)	(0.117)	(0.077)	(0.180)**	(0.164)
T3: Own health	0.071	0.120	-0.071	0.018	0.097	0.186
	(0.150)	(0.145)	(0.119)	(0.079)	(0.173)	(0.161)
T4: Public health	0.013	-0.025	-0.084	-0.030	0.081	0.148
	(0.155)	(0.148)	(0.120)	(0.083)	(0.175)	(0.164)
Observations	2,484	2,484	2,484	2,484	2,484	2,484
r2	0.7938	0.7994	0.9043	0.9662	0.5991	0.6952
meanT0End	4.8353	4.5969	5.8469	6.5717	3.2597	3.7752
T1vsT2	0.0000	0.0015	0.1352	0.2512	0.0000	0.0008
T1vsT3	0.0000	0.0033	0.5393	0.1424	0.0003	0.0019
T1vsT4	0.0000	0.0511	0.6091	0.0447	0.0005	0.0045
T2vsT3	0.8494	0.7792	0.3718	0.7321	0.1018	0.7680
T2vsT4	0.8525	0.2086	0.3141	0.3642	0.0897	0.6042
T3vsT4	0.7015	0.3191	0.9126	0.5710	0.9299	0.8165
State FE	Yes	Yes	Yes	Yes	Yes	Yes
Basic Controls	Yes	Yes	Yes	Yes	Yes	Yes

**Table 13:** Men: Impact of treatments on willingness to recommend COVID-19 vaccines (OLS)

Parameter	AZ	J & J	Moderna	Pfizer	Sinopharm	Sputnik-V
	(1)	(2)	(3)	(4)	(5)	(6)
T1: Scientific AZ debate	-0.487	-0.290	-0.029	-0.073	-0.515	-0.406
	(0.236)**	(0.243)	(0.192)	(0.123)	(0.284)*	(0.274)
T2: Misinformation	0.057	0.150	0.165	-0.030	0.514	0.348
	(0.229)	(0.235)	(0.184)	(0.113)	(0.299)*	(0.268)
T3: Own health	0.071	0.067	0.231	0.095	0.267	0.073
	(0.230)	(0.240)	(0.180)	(0.090)	(0.320)	(0.279)
T4: Public health	0.068	0.353	0.211	0.045	0.376	0.574
	(0.219)	(0.226)	(0.177)	(0.085)	(0.300)	(0.271)**
Observations	834	834	834	834	834	834
r2	0.8712	0.8590	0.9329	0.9813	0.6587	0.7648
meanT0End	5.3371	5.0857	5.9257	6.7371	3.4457	4.1771
T1vsT2	0.0261	0.0753	0.3220	0.7367	0.0002	0.0054
T1vsT3	0.0217	0.1558	0.1772	0.1400	0.0085	0.0860
T1vsT4	0.0177	0.0064	0.1969	0.2691	0.0013	0.0003
T2vsT3	0.9536	0.7316	0.7147	0.2101	0.4298	0.3170
T2vsT4	0.9626	0.3765	0.7974	0.4326	0.6379	0.3895
T3vsT4	0.9887	0.2214	0.9101	0.4950	0.7294	0.0679
State FE	Yes	Yes	Yes	Yes	Yes	Yes
Basic Controls	Yes	Yes	Yes	Yes	Yes	Yes

**Table 14:** Role of economic preferences on treatment effects (OLS)

Parameter	(1)	(2)	(3)	(4)	(5)	(6)	(7)
Panel A: Recommend AZ							
T1: Scientific AZ debate	-0.724	-0.725	-0.550	-0.724	-1.019	-0.735	-0.768
	(0.104)***	(0.104)***	(0.358)	(0.104)***	(0.286)***	(0.104)***	(0.160)***
Risk aversion		-0.014	-0.006				
		(0.032)	(0.036)				
T1 x risk aversion			-0.041				
			(0.081)				
Patience				0.004	-0.005		
				(0.016)	(0.018)		
T1 x patience					0.045		
					(0.041)		
Altruism						-0.018	-0.020
						(0.016)	(0.018)
T1 x altruism							0.011
							(0.040)
Observations	3,318	3,318	3,318	3,318	3,318	3,276	3,276
Panel B: Recommend Pfizer							
T1: Scientific AZ debate	0.066	0.065	0.030	0.065	0.093	0.065	0.063
	(0.052)	(0.052)	(0.179)	(0.052)	(0.143)	(0.052)	(0.080)
Risk aversion		-0.033	-0.034				
		(0.016)**	(0.018)*				
T1 x risk aversion			0.008				
			(0.041)				
Patience				-0.002	-0.001		
				(0.008)	(0.009)		
T1 x patience					-0.004		
					(0.020)		
Altruism						0.005	0.005
						(0.008)	(0.009)
T1 x altruism							0.001
							(0.020)
Observations	3,318	3,318	3,318	3,318	3,318	3,276	3,276
State FE	Yes	Yes	Yes	Yes	Yes	Yes	Yes
Basic Controls	Yes	Yes	Yes	Yes	Yes	Yes	Yes

*Notes:* ‘AZ’ refers to AstraZeneca and ‘Pfizer’ refers to Pfizer/BioNTech. State FE include controls for respondents place of residence (state). Basic controls include respondent’s age, gender, education, and survey specifics (order of vaccine-related questions, platform, device). Robust standard errors used. Standard errors are in parentheses. */**/*** denotes 10/5/1 percent significance levels

**Table 15:** Role of health experience on treatment effects (OLS)

Parameter	(1)	(2)	(3)	(4)	(5)	(6)	(7)
Panel A: Recommend AZ							
T1: Scientific AZ debate	-0.724	-0.720	-0.742	-0.723	-0.746	-0.724	-0.823
	(0.104)***	(0.103)***	(0.117)***	(0.103)***	(0.122)***	(0.104)***	(0.174)***
Risk group		-0.258	-0.279				
		(0.104)**	(0.116)**				
T1 x Risk group			0.102				
			(0.250)				
Received AZ				0.189	0.173		
				(0.092)**	(0.102)*		
T1 x Received AZ					0.083		
					(0.231)		
Received Pfizer						-0.062	-0.092
						(0.086)	(0.096)
T1 x Received Pfizer							0.154
							(0.217)
Observations	3,318	3,318	3,318	3,318	3,318	3,318	3,318
Panel B: Recommend Pfizer							
T1: Scientific AZ debate	0.066	0.066	0.057	0.063	-0.034	0.062	0.246
	(0.052)	(0.052)	(0.059)	(0.051)	(0.061)	(0.051)	(0.086)***
Risk group		-0.001	-0.009				
		(0.052)	(0.058)				
T1 x Risk group			0.037				
			(0.125)				
Received AZ				-0.285	-0.351		
				(0.046)***	(0.051)***		
T1 x Received AZ					0.346		
					(0.115)***		
Received Pfizer						0.288	0.343
						(0.043)***	(0.047)***
T1 x Received Pfizer							-0.286
							(0.107)***
Observations	3,318	3,318	3,318	3,318	3,318	3,318	3,318
State FE	Yes	Yes	Yes	Yes	Yes	Yes	Yes
Basic Controls	Yes	Yes	Yes	Yes	Yes	Yes	Yes

*Notes:* ‘AZ’ refers to AstraZeneca and ‘Pfizer’ refers to Pfizer/BioNTech.State FE include controls for respondents place of residence (state). Basic controls include respondent’s age, gender, education, and survey specifics (order of vaccine-related questions, platform, device). Extended controls refers to additional controls on respondent’s preferences (risk, patience, altruism) and whether she/he had ever been diagnosed with Covid. Robust standard errors used. Standard errors are in parentheses. */**/*** denotes 10/5/1 percent significance levels

**Table 16:** Impact on satisfaction with government institutions (OLS)

	Government		Ministry of Health		Minister
Parameter	National	State	National	State	National
	(1)	(2)	(3)	(4)	(5)
T1: Scientific AZ debate	0.075	0.107	0.067	0.083	-0.005
	(0.048)	(0.051)**	(0.048)	(0.047)*	(0.047)
T2: Misinformation	0.012	-0.007	-0.015	0.075	-0.031
	(0.048)	(0.050)	(0.048)	(0.047)	(0.047)
T3: Own health	0.005	0.016	0.019	0.054	-0.047
	(0.048)	(0.050)	(0.047)	(0.046)	(0.046)
T4: Public health	0.103	0.013	0.064	0.060	0.015
	(0.048)**	(0.050)	(0.047)	(0.046)	(0.047)
Observations	3,015	3,015	3,015	3,015	3,015
State FE	Yes	Yes	Yes	Yes	Yes
Basic Controls	Yes	Yes	Yes	Yes	Yes

*Notes:* ‘AZ’ refers to AstraZeneca. State FE include controls for respondents place of residence (state). Basic controls include respondent’s age, gender, education, and survey specifics (order of vaccine-related questions, platform, device). Robust standard errors used. Standard errors are in parentheses. */**/*** denotes 10/5/1 percent significance levels

**Table 17:** Impact on perceptions of future (behaviour) (OLS)

	Living conditions			Health-related behaviour	
Parameter	No Covid	Easy travels	Open bars	Covid-App	DPT vaccine
	(1)	(2)	(3)	(4)	(5)
T1: Scientific AZ debate	0.050	0.003	0.094	0.023	0.127
	(0.080)	(0.088)	(0.090)	(0.053)	(0.090)
T2: Misinformation	0.113	0.090	0.120	-0.019	0.162
	(0.079)	(0.088)	(0.090)	(0.053)	(0.090)*
T3: Own health	-0.015	-0.023	0.009	-0.062	0.056
	(0.078)	(0.087)	(0.088)	(0.052)	(0.089)
T4: Public health	-0.084	-0.012	-0.057	-0.022	0.070
	(0.079)	(0.088)	(0.089)	(0.052)	(0.090)
Observations	3,173	3,173	3,173	3,289	3,264
State FE	Yes	Yes	Yes	Yes	Yes
Basic Controls	Yes	Yes	Yes	Yes	Yes

*Notes:* ‘AZ’ refers to AstraZeneca. State FE include controls for respondents place of residence (state). Basic controls include respondent’s age, gender, education, and survey specifics (order of vaccine-related questions, platform, device). Robust standard errors used. Standard errors are in parentheses. */**/*** denotes 10/5/1 percent significance levels

## Data Availability

All data used in this study are available at https://osf.io/6fnye/. A copy of the materials used in this study, as displayed to respondents, can be obtained from the authors upon request. Source data are provided with this paper. Furthermore, the statistical code to run all data cleaning steps and the analysis for this study is available at https://osf.io/6fnye/.

## Footnotes

1 The “first wave” was in March/April 2020 with about 6,000 new cases per day, while the “second wave” was in October 2020 until January 2021 with up to 32,000 new cases per day. The situation improved in February to March 2021 with cases substantially rising again in April 2021.

2 Vaccines were officially approved in Germany in the following order: 1. Pfizer/BioNTech on 21 December 2020, 2. Moderna on 6 January 2021, 3. AstraZeneca on 29 January 2021, and 4. Johnson & Johnson on 11 March 2021.

3 Since health workers belonged to priority groups 1 & 2 they gained preferential access to COVID-19 vaccines. In mid-April 2021 about 32.4 percent of nurses were fully vaccinated and another 44.6 percent of nurses had already a first injection [16]. From the start of the vaccination campaign in December 2020 until today health workers have not been mandated by the government or employers to become vaccinated. In many cases, however, employers (hospitals, practices) have asked their staff to become vaccinated against COVID-19.

4 Efficacy rates refer to the ALPHA variant of SARS-CoV-2.

5 While in the case of Pfizer/BioNTech vaccinations were recommended for the age group 18 years and older consistently since December 2020, official recommendations for AstraZeneca changed multiple times. Germany’s Permanent Vaccine Commission (Ständige Impfkommission) initially suggested on 4 February 2021 to ideally use the vaccine for the age group 18 to 64 only [17], it revised the recommendation on 12 March 2021 to include everyone older than 17 years [18], and ultimately suggested on 1 April 2021 that the vaccine is best suited for individuals older than 59 years [19].

6 While most of the literature on information experiments assumes estimated treatment effects to be causal once covariate balance is achieved, we consider estimates to be associative in nature. There are two reasons for this: First, randomization might still have resulted in imbalances in unobservables. Second, in our context effects are more difficult to interpret as causal given the circumstance that health workers had been exposed for already many months to different COVID-19 vaccine-related information prior to our intervention. Thus, it is likely that the information provided in the treatments was not new to the group of health workers, and the experimental manipulation likely made only a piece of specific information more salient. For the experiment, we derive a minimum detectable effect size (MDE) of about 6.6 percentage points. Calculations assume a statistical power of 80 percent at a significance level of 5 percent.

7 X comprises the following variables: age, gender, education, vaccine order, social media platform, and device type.

8 A possible concern with respect to the impact of T1 on the willingness to recommend the vaccine of AstraZeneca relates to experimenter demand effects. We believe that the observed spillover effects provide credible evidence on the circumstance that health workers’ reactions to ambigious signals with respect to a vaccine are not AstraZeneca specific and do neither reflect experimenter demand effects nor social desirability bias.
